# Determination of Composition of Masonry Mortars for Conservation of Historical Constructions Using Artificial Neural Networks

**DOI:** 10.3390/ma18163851

**Published:** 2025-08-17

**Authors:** Filip Chyliński, Piotr Kupisz, Przemysław Więch, Lesław Brunarski

**Affiliations:** Instytut Techniki Budowlanej, Filtrowa Str. 1, 00-611 Warsaw, Poland; p.kupisz@itb.pl (P.K.); p.wiech@itb.pl (P.W.); l.brunarski@itb.pl (L.B.)

**Keywords:** artificial neural network, masonry mortar, microstructure, porosity, composition

## Abstract

This study presents a novel approach to determine the composition of masonry mortars and their types from cement, lime, and cement–lime using an artificial neural network (ANN). It also allows the preparation of mortar recipes for the conservation of historical masonry objects with properties similar to the original ones, but using currently available raw materials. An ANN was trained using a set of cement, lime, and cement–lime mortars with known compositions. The properties chosen for the ANN’s analysis included total porosity, specific density, insoluble residue content, silicone (SiO_2_) content, calcium (CaO) content, Si/Ca ratio in grout, and compressive strength. The use of ANNs allows for the determination of mortar composition with a validation error of less than 5% and a method of classification of the type of mortar that gives correct answers in more than 93% of cases, proving the usefulness of ANNs in determining the type and composition of masonry mortars relevant for the conservation of historical masonry structures.

## 1. Introduction

Mortar is a binder used to bond masonry elements in masonry structures. The mortar permanently bonds the masonry elements and transfers loads to the entire masonry structure. The compressive strength of the mortar was slightly lower than that of the masonry elements. This allows a slight deformation of the mortar and prevents cracking of the masonry elements when local overloading occurs [1,2]. The use of mortars with significantly higher strengths than those of masonry elements is unacceptable because it does not ensure the proper operation of the masonry structure. However, the use of mortars with extremely low strengths weakens the entire structure [3]. Therefore, when performing repair work on masonry structures, it is crucial to reproduce the original mortar as accurately as possible. An additional aspect that may arise is working with historic structures, where the properties of the mortar and its type should be reproduced as faithfully as possible in relation to the original structure [4,5]. In this type of investigation, first of all, the type of mortar needs to be compatible with the original, and the properties need to be as close as possible to the original; however, composition might vary due to the major changes in the properties of binder materials (cement and lime). Working with masonry structures makes it impossible to take a mortar sample large enough to perform tests such as compressive strength tests according to standards, where 40 mm × 40 mm × 160 mm samples are required. Therefore, it is necessary to define the properties of mortar based on its composition. The components of the analysed mortars were Portland cement, lime, and fine aggregates. The amount of water was not considered because it is usually dosed in an amount appropriate to the desired consistency, which depends mainly on the type of ingredients used, their specific surface area, and water demand. Examining only the elemental composition of the hardened mortar does not provide sufficient information because the main elements in mortar are calcium, silicon, aluminium, and iron, which are also present in each of the mortar ingredients used. Therefore, several other determinable properties of hardened mortar must be investigated to determine possible correlations with the composition.

Another issue in determining the composition of masonry mortars might occur during the conservation of historical objects because the original mortars were cast using historical constituents made using different raw materials [6]. In addition, the details of the production and technology differ [4]. The finest binders used were significantly lower, which further determined mortar properties. This brings us to a crucial question: should the new mortar have the same composition as the original historical mortar, or should it have similar properties? Using constituents in the same proportions as in historical mortars, we would obtain a mortar with a higher compressive strength and lower porosity. As the compressive strength of the mortar should not be higher than that of the masonry unit, it can be concluded that the properties of the new mortar should be similar to those of the original mortar, and the composition is not critical. The type of mineral binder should be the same as that of the original, owing to historical compatibility.

Samples that might be collected from restored masonry objects are rather small, with a volume of approximately a few cubic centimetres. It is impossible to determine the full-scale compressive strength according to EN or ASTM standards [7,8]. Several other important properties, which might help describe a new mortar for renovation, such as porosity, density, chemical composition, and insoluble residue content, might be determined.

An artificial neural network (ANN) may be a useful tool for analysing the dataset obtained from the tests. ANNs are among the pillars of modern artificial intelligence, and their applications in various fields of science and industry are gaining importance. Built on a model of the human brain, an ANN can solve complex problems that traditional algorithms cannot solve efficiently. In recent years, there has been a noticeable increase in interest in the use of artificial neural networks in the construction industry, where their potential for data analysis, design process optimisation, and construction management has become invaluable [9,10,11]. The application of ANNs in building engineering covers a wide range of problems, including the prediction of loads on structures, forecasting of material consumption, and automatic design of building structures. Neural networks can support decision-making at every stage of the construction life cycle, leading to increased efficiency, cost reduction, and improved work quality. In addition, owing to their ability to learn from large datasets, ANNs can analyse and optimise construction processes that were previously difficult to predict using traditional analytical methods [12].

In recent years, artificial neural networks have gained popularity in the field of construction materials, including the design and optimisation of cementitious composites [13,14]. The composition of these materials is crucial for their mechanical properties, durability, and sustainability. Traditional methods for selecting the proportions of components, such as experimental studies or numerical methods, often require considerable time and funding. Consequently, the use of ANNs in the analysis and prediction of the composition of cementitious composites is becoming increasingly promising [15,16]. Molero et al. [17] used an ANN to determine the ratio of cement to sand in a mortar based on the results of non-destructive tests with ultrasonic techniques. Saridemir et al. [18] used an ANN to predict the long-term mechanical properties of cement composites with granulated blast furnace slag. The results of their research allowed the development of a method for determining the final compressive strength of composites without the need for a long-term maturation process, which is time-consuming. Saridemir [19] also used an ANN to determine the compressive strength of cement mortars with the addition of metakaolinite based on their composition, without the need for further performance or prolonged maturation. Asteris et al. [20] and Razavi et al. [21] dealt with the determination of the compressive strength of mortars using ANNs based on the knowledge of their composition. Terzic et al. [22] were using the ANNs to determine the performance of mortars with different types of cement and mineral additives. The above publications are thematically the most similar, among the others found, to the subject of this paper. It describes the process of predicting the compressive strength of mortars based on their composition using an ANN, while the purpose of this paper is to do the reverse, that is, to determine the composition based on the properties. Most investigated studies focused on predicting mechanical properties, mainly compressive strength, using ANNs and taking as input a dataset describing their composition. The novelty of the presented work is based on the use of ANNs to predict the type and composition of masonry mortars based on their properties. Another advantage is that the proposed method was developed to allow the use of small samples for tests, as only those are possible to collect from historical masonry buildings.

The aim of this study was to determine the composition of mortar with characteristic features consistent with empirical values. This study included the development of a database containing a set of characteristic features and reference mortars and then subjecting them to analysis using artificial neural networks. The proposed concept for determining mortar composition is novel because it is based on data from reference composites, whereas the dataset for the tested unknown mortar is relatively small. The proposed method was developed in a way that allows it to be used in real masonry and historical structures.

## 2. Materials and Methods

Preliminary tests with unpublished results involved a large number of tests, including volume density, water absorption, total porosity, macropore content, micropore content, nanopore content, compressive and flexural strength, phase composition, and elemental composition (SiO_2_, CaO, Al_2_O_3_, and Fe_2_O_3_). There are many other important properties of mortars, especially for the conservation of historical, old buildings, such as capillarity, water vapour permeability, and modulus of elasticity; however, it is impossible to perform standard tests because of the sizes of samples collected from the masonry construction. The use of a multi-criteria analysis of the test results helped limit the number of important characteristics that should be examined to reconstruct the composition of the original mortar. The basic characteristics examined and presented in this study are as follows:

- Insoluble residue content;
- Total porosity;
- Specific density;
- Silicon content (SiO_2_);
- Calcium content (CaO);
- Molar ratio of Si/Ca in the grout;
- Compressive strength (as an additional characteristic).

The chosen basic input characteristics for further ANN investigations should depend on the mortar composition; however, they should be as independent of each other as possible, and one characteristic should not simply imply the other. The insoluble residue content is the amount of mortar that did not react with hydrochloric acid. This is related to the CaO content, as the Ca compounds are mostly soluble, and SiO_2_ is mostly insoluble in HCl. However, the results have shown that these characteristics are different from the CaO and SiO_2_ content determined by the XRF method and cannot be simply calculated from one another. Si/Ca ratio determined by examining the grout area using the SEM-EDX technique provides another set of independent chemical information related to the composition of mortars, as these analyses are performed only on the grout without aggregate grains.

The total porosity and specific density provide quite independent forms for each other ’s data sets. The total porosity provides information about the microstructure and total air voids in the mortar, and the specific density tests are carried out on ground samples of mortar and provide average information about the physical parameters of grout and aggregates without air voids.

Owing to the nature of the test samples, which are often taken from masonry structures, it may not be possible to test their compressive strength; therefore, this performance was not considered in the ANN analysis. The obtained values of the test results of the reference mortars with known compositions made it possible to train artificial neural networks. Two types of neural networks were prepared: classification and quantitative. The task of the classification networks is to determine the type of mortar from cement, cement–lime, and lime. The role of a quantitative ANN is to determine the proportion of the constituents used to prepare the mortar.

### 2.1. Materials

The compositions of the reference mortars were selected in accordance with the requirements of PN-B-10104, Polish national standard [23], and ASTM C270-10 [24]. Portland cement CEM I 42.5R in accordance with EN 197-1 [25], lime CL 90-S in accordance with EN 459-1 [26], and river sand were used for the preparation of mortar samples. Table 1 lists the elemental compositions of the constituents used for casting. The composition was determined using WD-XRF (X-ray fluorescence spectroscopy with wave diffraction), (Bruker AXS, Karlsruhe, Germany). The samples were melted with a flux before measurement.

The compositions of the reference mortars are listed in Table 2. The volume proportions rejection in the standard were converted into mass proportions after determining the bulk densities.

### 2.2. Methods

To achieve the aim of this study, the following characteristics were determined:

- Total porosity;
- Specific density;
- Insoluble residue content;
- Silicon content (SiO_2_);
- Calcium content (CaO);
- Molar ratio of Si/Ca in the grout;
- Compressive strength (as an additional characteristic).

#### 2.2.1. Total Porosity

The total porosity and pore size distribution were determined using mercury porosimetry. However, only the total porosity was found to be useful for further ANN analyses. The test was carried out on drilled cores approximately 25 mm in diameter and 35 mm in height, taken from larger cylindrical samples 160 mm in diameter and 60 mm in height, after maturation for 28 days. The cut specimens were first dried at 40 °C for 24 h and then under reduced pressure (20 mbar) for 4 h at room temperature to remove the maximum amount of moisture. The dried samples were immersed in anhydrous ethanol under a reduced-pressure atmosphere to stop hydration and were stored until testing. Immediately before the test, the samples were removed from ethanol and dried, as described above, at 40 °C and then under reduced pressure. The pore distribution test was performed using a mercury porosimeter (Quantachrome Poremaster model PM608, Anton Pear Group, Boynton Beach, FL, USA), which allowed the analysis of the distribution of pores with diameters greater than 3.6 nm. Each test was performed on three samples, and the average value was calculated.

#### 2.2.2. Specific Density

The specific density of the hardened mortars was determined using the pycnometric method on mortar samples ground to grains with diameters less than 0.125 mm, in accordance with EN 1097-7:2023-04 [27]. Each test was performed on three samples, and the average value was calculated.

#### 2.2.3. Insoluble Residue Content

The insoluble residue content was determined according to EN 196-2:2013-11 [28]. The test samples were dried at 40 °C and ground into grains smaller than 0.125 mm. Two determinations of the content of insoluble parts were performed for each mortar, and the average value was calculated according to the requirements of the standard.

#### 2.2.4. Silicone (SiO_2_) and Calcium (CaO) Content

Silicon (SiO_2_) and calcium (CaO) contents were determined using X-ray fluorescence spectroscopy with a wave diffraction (WD-XRF) model Jaguar S6 produced by Bruker (Bruker Nano GmbH, Berlin, Germany). Samples of approximately 10 g of mortar were prepared by crushing and grinding to a particle size of <0.063 mm. Subsequently, they were ignited, and their loss on ignition (LOI) was determined. Samples after ignition were used to prepare fused samples using as a flux a mixture of 66.67% Li_2_B_4_O_7_, 32.83% LiBO_2_, and 0.5% LiBr.

#### 2.2.5. Si/Ca Ratio in Grout

**Figure 1:**
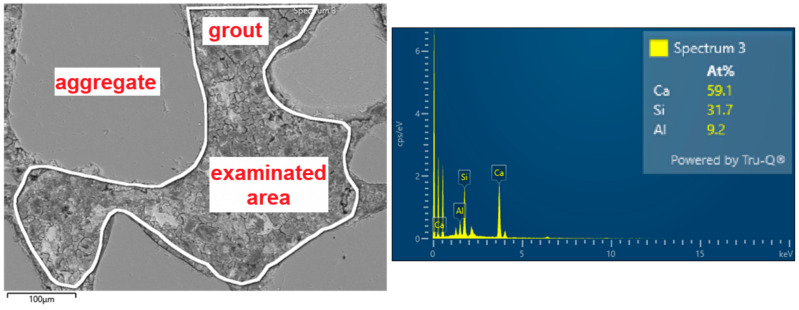
Example of analysis of the area of grout from the mortar.

The polished sections were immersed in resin from each type of mortar. The details of the sample preparation are described in a previous publication [29]. The elemental composition of the grout (without aggregates) in the micro area of the mortars was analysed using a Sigma 500 VP scanning electron microscope (SEM) manufactured by ZEISS (Carl Zeiss Microscopy GmbH, Köln, Germany). The elemental composition was determined using an UltimMax 40 X-ray energy-dispersive X-ray detector (EDX) produced by Oxford Instruments (Oxford Instruments, High Wycombe, UK). For each type of sample, ten random areas were investigated, and molar compositions including only Ca, Si, and Al were determined. The average Si/Ca ratio was determined from the obtained data. Figure 1 presents an example of the analysed area of mortar with a marked area of grout whose composition was determined.

#### 2.2.6. Compressive and Flexural Strength

The compressive and flexural strengths of the reference mortars were determined in accordance with EN 1015-11:2020-04 [7]. Each test was carried out on three 40 mm × 40 mm × 160 mm specimens, yielding six results for compressive strength and three for flexural strength. Samples were matured for 28 days before testing at a temperature of 20 ± 2 °C and humidity depending on the type of mortar (cement mortars in >95%, cement–lime mortars for 7 days in >95% and later in 60 ± 5%, and lime mortar in 60 ± 5%).

#### 2.2.7. ANN Analysis

**Figure 2:**
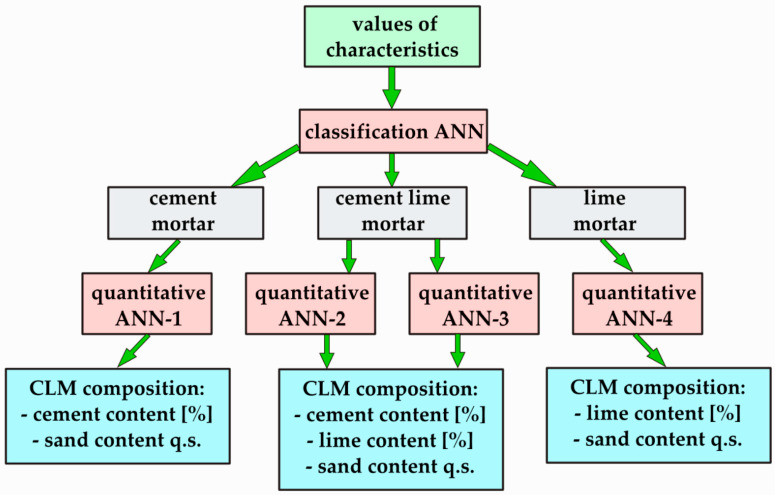
Scheme of composition determination using ANNs.

The results obtained from the reference mortars were analysed using ANNs. Two types of ANNs were developed: classification and quantitative ANNs. Four separate quantitative ANNs were prepared: one for CM and LM, and two for CLM prediction. ANN analyses were performed using Statistica. Statistical software package (StatSoft GmbH, TIBCO Software Inc. ver. 13, Palo Alto, CA, USA) were used in this study for data analysis. The adopted methodology assumes that the unknown mortar will first be classified, and then its material composition will be determined according to the scheme presented in Figure 2.

## 3. Results and Discussion

The results of the tests were analysed in the scope of their usefulness in further proceedings using an ANN. As a first step, a linear correlation between the binder content and the values of the analysed performance was determined using the regression value (square of the coefficient of multiple correlations, R^2^). To evaluate the sensitivity of each correlation, the value of the directional coefficient of a linear function was determined as tgα, where α is the angle of inclination of the line. The higher the absolute value of the directional coefficient, the more sensitive the correlation is. Analyses were performed separately for the sets of data for each mortar type. Different weights for each property were established using Statistica software, and an initial set of ANNs was generated. After their analysis, a few ANNs were chosen based on their properties (e.g., validation error) and further usability. A sensitivity analysis was conducted to test the significance of the mortar properties.

### 3.1. Total Porosity

**Figure 3:**
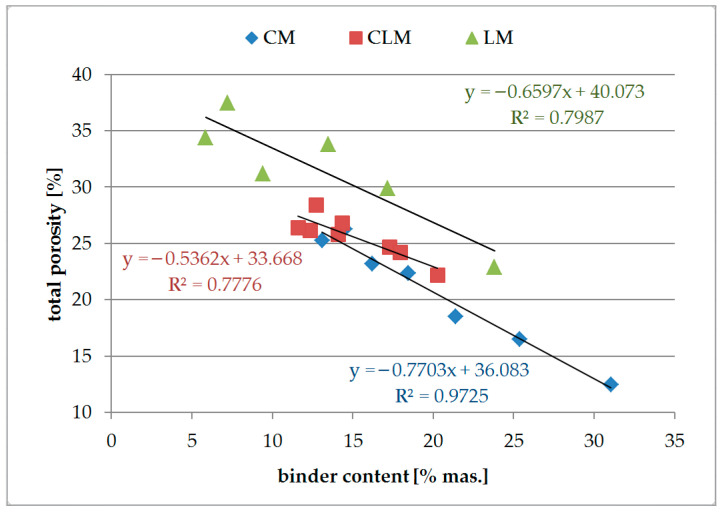
Total porosity.

During the analysis of the results obtained using MIP, the pore size distribution for each type and composition of mortar was analysed. However, sufficient correlations were found to be promising as a characteristic of the properties as a function of binder content. Only the total porosity values appear promising for further ANN analyses. Figure 3 shows the total porosity of the analysed mortars as a function of the binder content.

Analysis of the results of the total porosity test of the tested mortars showed that an increase in the binder content decreased the total porosity for each type of mortar. The highest values of porosity were obtained for lime mortars, and the lowest for cement mortars. It must be established that no air-entraining admixtures were added to the tested mortars. Such admixtures might be required to obtain mortars with high porosity. In such cases, the dataset for ANN training should be extended to include reference mortars.

The correlation between the total porosity of the mortar and binder content was found to be the best for the CM samples, where R^2^ = 0.97, and the absolute value of the directional coefficient of the linear function had a value of approximately 0.8, which should result in sufficient sensitivity. Total porosity is a useful property of CM for further investigation. For the CLM and LM samples, the R^2^ values were lower than those for CM. Although correlations in the range of approximately 0.79–0.80 might be found to be useful for further examinations using ANN, they were not rejected at this step. The values of the directional coefficients were also within the accepted range of 0.5–0.7.

### 3.2. Specific Density

**Figure 4:**
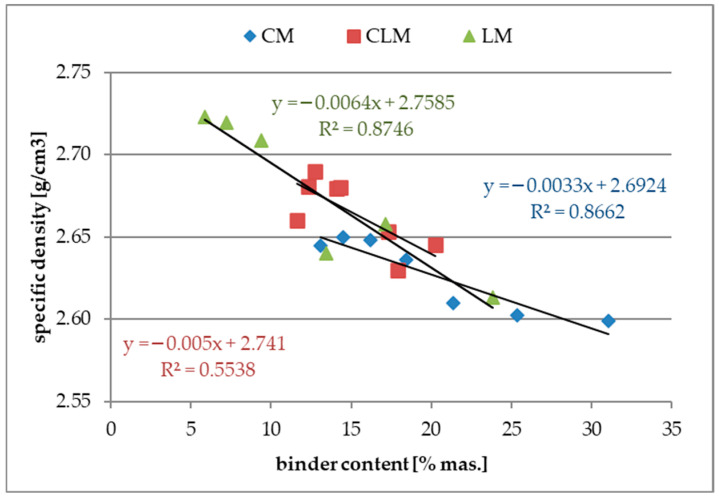
The specific density of mortars.

Figure 4 shows the results of the specific density tests as a function of the binder content.

An increase in the binder content in all types of binders caused a slight decrease in their density. This relationship seems to be strange as the binder, especially cement, has a higher density than sand. In addition, the results of the total porosity tests showed a decrease in this feature by increasing the binder content, which should affect the increase in density. This observation might be explained by the increase in large macropores with an increase in binder content, and those macropores are out of the range of the MIP method.

Analysing the plots presented in Figure 4, a correlation of approximately 0.87 for CM and LM was observed. However, the linear coefficients for all three functions had very low values (less than 0.01), indicating the weak sensitivity of this property for further investigations. However, the uncertainty of the test method is at the level of about ±0.001 g/cm^3^, and these results might still be useful. The correlation coefficient for the CLM samples was very low (<0.6), indicating a very weak correlation. One of the causes of this observation may be related to the binder content, which was strictly defined as the sum of the cement and lime contents without any weights or coefficients, as they are not equal binders. All the results of the tests for specific density were further analysed using ANNs.

### 3.3. Insoluble Residue Content

**Figure 5:**
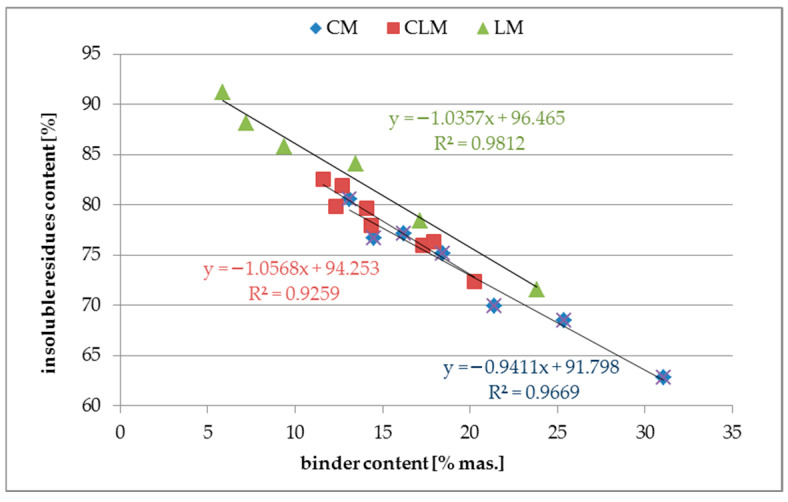
Insoluble residue content in mortars.

Figure 5 shows the results of the insoluble residue tests as a function of the binder content.

The insoluble residue content in typical quartz sand used for mortars is very high, and in most cases, it is approximately 90–95%. For most binders, this feature is relatively low; for example, for OPC, it is approximately 5%. This leads to the conclusion that an increase in the binder content will decrease the insoluble content in mortars. Such a relationship was observed in all types of tested mortars.

Analysis of the results of insoluble particle tests for mortars showed a very high correlation R^2^ in the range from 0.93 for CLM to 0.98 for LM samples. The linear coefficients for each of the three functions had an absolute value of approximately one, indicating an acceptable sensitivity of this property for further ANN investigations in the future. The high correlation, even for CLM, might be caused by the fact that the cement and lime used have very low values of insoluble particle content (less than 5%). In such cases, the insoluble part content is highly related to the sand content of the mortar.

### 3.4. Silicon (SiO_2_) and Calcium (CaO) Content

**Figure 6:**
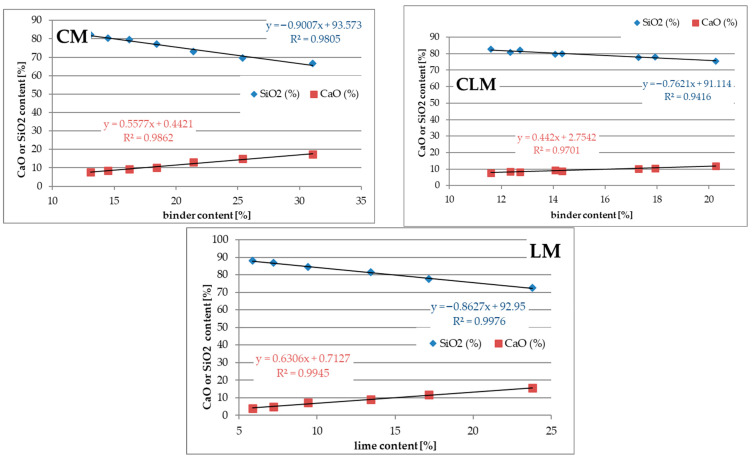
Silicon (SiO_2_) and calcium (CaO) content.

The main elements of each mortar were determined using WD-XRF spectroscopy. However, in most cases, no significant correlation was found between the elemental and binder contents. Only the analysis of Si (SiO_2_) and Ca (CaO) content is promising for further investigation. Figure 6 presents a set of plots showing the elemental content as a function of the binder content. The CLM data were separated into two sets of plots: one for the cement content and the other for the lime content.

Analysis of the results of the SiO_2_ and CaO content tests showed that an increase in the binder content in all types of mortars decreased the SiO_2_ content and increased the CaO content. This may be explained by the fact that SiO_2_ is the main constituent of quartz sand (75%) and a minor constituent of cement (19%) and lime (1.5%). CaO content increased in all types of tested mortars with an increase in the binder content. This is caused by the high CaO content in cement and lime (62% and 70%, respectively) and the low CaO content in sand (8%).

By analysing the correlations between SiO_2_, CaO, and binder contents, it was found that there was a good correlation between CM, LM, and CLM for both constituents. R^2^ values were greater than 0.94. The linear coefficients for all analysed plots were in the range of 0.5 to 1.3, except for the CaO plot for CLM as a function of cement content, where a value of 0.4 was obtained. In most cases, the sensitivity of these functions is sufficient for further investigation.

### 3.5. Si/Ca Ratio in the Grout

**Figure 7:**
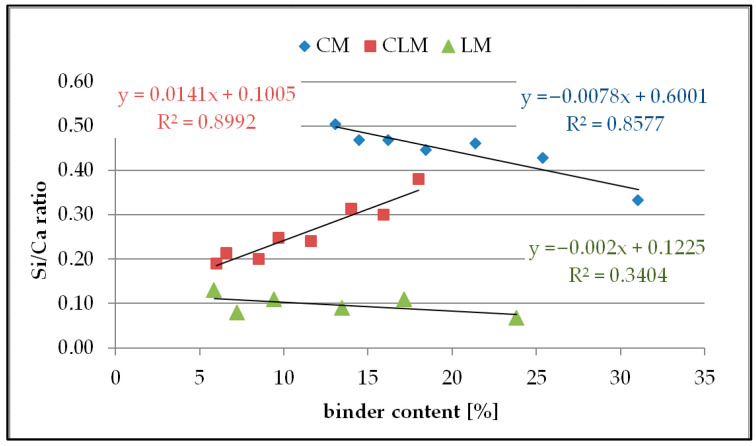
Si/Ca ratio in the grout.

Figure 7 shows plots of the Si/Ca ratio in the grout as a function of the binder content for each type of mortar.

The results of the received Si/Ca values in the grout as a function of the binder content showed differences for each type of mortar, including the different monotonicity of the function. The highest values for Si/Ca were observed for the CM samples because of the relatively high silicone content in the grout from the Portland cement hydration products and relicts. An increase in cement content causes a decrease in Si/Ca owing to the higher saturation of the grout with Ca ions.

In CLM, a different monotonicity was observed compared to CM. The increase in binder content causes an increase in the Si/Ca ratio, which may be caused by the various proportions of cement and lime in the binder, which affects the grout composition.

The Si/Ca ratio values in LM were not very diverse, which might be caused by the rapid saturation of grout with CaO, and further increases in lime content did not cause significant changes in grout.

Analysing the correlation for each type of mortar, it can be seen that CM and CLM have a good linear correlation, reaching R^2^ values of approximately 0.86 and 0.90, respectively. However, the R^2^ value for LM was very low (0.34), indicating no linear correlation. The linear coefficients for each of the analysed functions had low absolute values, showing the rather weak sensitivity of this method, especially for LM, where the value of this coefficient was approximately 0.002 g.

### 3.6. Compressive and Flexural Strength

Table 3 presents the results of the flexural and compressive strength tests. The flexural strength of LM was not recorded because of its very low values.

The compressive strength of the tested mortars was in the range of 8 ÷ 43 MPa for CM, 2 ÷ 19 MPa for CLM, and 0.09 ÷ 0.18 MPa for LM. The mechanical properties of the tested mortar cover the entire range typically used in masonry and historical constructions. The mechanical properties were not further investigated using ANNs. Their role was to establish a range of mortar properties, where the proposed method for determining the composition might be useful.

### 3.7. Artificial Neural Network Analysis

#### 3.7.1. ANN—Classification

The purpose of the ANN classification was to determine the type of mortar from cement, cement–lime, and lime based on the determined values of the physical and chemical characteristics. Table 4 presents the data used for further examination with ANN.

Table 5 lists the basic characteristics of the input data used in this study.

The analysed data were randomly divided into the following groups according to the proportions given:

- Learning, 70%;
- Testing, 15%;
- Validation, 15%.

During the analyses and subsequent iterations, the following ANNs were selected, the basic characteristics of which are listed in Table 6.

The selected classification ANNs are characterised by a varying number of neurons in the hidden layer and use a different activation function for the neurons in the hidden layer. This results in observed changes in the quality of learning and testing. ANN No. 2 exhibited the best testing quality, and ANN No. 3 exhibited the highest learning quality. All ANNs were further analysed. Table 7 presents a summary of the confusion matrices for the analysed ANNs.

By analysing the confusion matrix for the selected ANNs presented in Table 7, a variation in terms of correctness in the assessment of the type of mortar under investigation based on the values of the input data can be observed. For the determination of the type of mortar among cement, cement–lime, and lime, the best results were obtained for ANN No.3, which provided a typing accuracy of 93%, which is a very good result, demonstrating the potential usefulness of this method. The ANNs were subjected to a sensitivity analysis of the learning samples. Table 8 presents the results of these analyses.

The sensitivity analysis of the selected ANNs showed a moderate level of sensitivity for ANN Nos. 1 and 2, close to unity for each of the input parameters. In contrast, the sensitivities of SNF No. 3 were characterised by much wider ranges of values, exceeding the value of eight for the ‘total porosity’ attribute. From this analysis, it can be seen how significantly the individual components influence the outcome of the ANN analysis. The total porosity, insoluble residue content, SiO_2,_ and CaO are important. In contrast, the specific density and Si/Ca ratio in the grout were parameters of lesser importance for ANN No. 3. This conclusion proves the initial analysis of the specific density and Si/Ca ratio as values with lower importance than others (p. 3.2 and p. 3.5)

ANN No. 3 is an example of an artificial neural network that can be used for the classification of mortar types.

#### 3.7.2. ANN—Quantitative

To determine the material composition of the mortar to be tested, it is first necessary to classify the mortar using an ANN into one of the following types:

- Cement mortar;
- Lime mortar;
- Cement–lime mortar.

The next step was to determine the cement or lime content of cement and lime mortar. The remainder of the composition was a fine aggregate. Water content was not included in the composition. When determining the composition of the cement–lime mortar, the cement content was determined using an ANN, and the lime content was determined using a different ANN. The remainder of the composition was a fine aggregate. It is possible to develop a single ANN to determine the cement and lime contents simultaneously, but this usually yields worse results than the two separate networks.

ANN quantitative—cement mortars

Based on these analyses, five different ANNs were selected for further verification. The basic parameters are listed in Table 9.

A preliminary evaluation of the parameters of the obtained networks showed that ANN No. 5 had the best learning quality; however, it also had the highest testing errors. This may be because the network has mastered the input dataset very well by matching it fully; however, this carries the risk of very limited versatility in processing data other than that of the initial dataset. ANN No. 1 exhibited a relatively high testing error. Therefore, ANN Nos. 1 and 5 were excluded from further analysis. The most promising ANNs among those analysed were ANN Nos. 2, 3, and 4. The sensitivity analyses of the networks are presented in Table 10.

Sensitivity analysis of the selected ANNs showed a very high variation in this characteristic among the different ANNs. The ANN with the most varied sensitivity was SSN No. 3. The sensitivity analysis presented in this study can be particularly useful in selecting a suitable network when there are incomplete analyses of an unknown mortar. In this case, the network with the lowest sensitivity should be selected.

ANN quantitative—lime mortars

Based on the analyses conducted, five ANNs for lime mortar were pre-selected, and their basic parameters are listed in Table 11.

Based on a preliminary parameter analysis of the obtained SSNs, SSN No. 2 and SSN No. 5 were rejected owing to their high values of validation and testing errors, respectively. The remaining networks were subjected to global sensitivity analysis. Table 12 presents the results of this study.

The results of the sensitivity analysis, as presented in Table 12, indicate similar properties for all ANNs. Further typing of a suitable network can be performed by selecting an ANN with a relatively small number of neurons in the hidden layer, owing to the small size of the learning dataset. Therefore, ANN No. 3 was the most suitable for this application.

ANN quantitative—Cement–Lime Mortars

Two types of ANNs were developed to determine the cement and lime contents separately. This approach provides better control of the network parameters than a single ANN, providing two parameters simultaneously in response, resulting in more reliable simulation results. Table 13 lists the pre-selected ANNs for determining the cement content of the cement–lime mortars.

The preliminary analysis of the selected ANNs allowed the rejection of ANNs 1, 4, and 5 because of their relatively high validation error values. The remaining networks were subjected to a global sensitivity analysis. The results of this assessment are shown in Table 14.

The sensitivity analysis results showed a similar range for both ANNs. However, ANN No. 2 was the most suitable network because of the smaller number of neurons in the hidden layer.

Table 15 lists the basic parameters of the pre-selected networks for determining the lime content of the cement–lime mortar.

The results of the sensitivity analysis indicated relatively low error values and the best learning quality. The other networks had similar sensitivity levels, except for ANN No. 5, which had a high validation error. The most promising networks in terms of application seem to be SSN No. 1 and SSN No. 4 because of the small number of neurons in the hidden layer in relation to the size of the test set. This may affect the versatility of the proposed network.

ANN limitations

The results of the tests and analyses presented in this study show that ANN may be a very useful tool for determining the composition of masonry mortars. However, this method has some limitations. The main limitation comes from the set of data that is used for learning, testing, and validation. Two aspects need to be considered: dataset size and the range of analysed values of properties. The compressive strengths of the mortars investigated in this study were in the range of 8–43 MPa, 2–20 MPa, and 0.09–0.13 MPa for cement, cement–lime, and lime mortars, respectively (Table 3), which cover most of the widely used masonry mortars. However, if the analysis of new, unknown mortar taken from conserved masonry buildings shows results of some property that is out of the range of reference mortars that were tested (e.g., total porosity), the set of reference data should be extended, and new ANNs should be developed.

## 4. Conclusions

Recovering the composition of masonry mortar in conserved mortar is a very important and challenging task that needs to be performed properly, especially in historical buildings to prolong their service life. However, the composition of the new mortar should be compatible with the type and properties of the original mortar, which will not always lead to the same composition owing to the different raw materials used today.

Analyses of tests performed on masonry mortars have led to the conclusion that it is possible to determine the type and composition of historical masonry mortars by examining a set of tests on small samples that may be collected from masonry construction. The set of tests that must be performed includes the following:

- Total porosity;
- Specific density;
- Insoluble residue content;
- Silicone (SiO_2_) content;
- Calcium (CaO) content;
- Si/Ca ratio in the grout.

The range of values of the properties of the tested mortars used as the dataset covered most of the presently and historically used masonry mortars. However, if an unknown mortar is investigated, it will be characterised by the values of properties which are outside the range of the used dataset; thus, a new reference mortar should be tested and added to the set, and new ANNs should be developed. Otherwise, extrapolation data from the dataset might cause an increase in errors, and the results might be insufficient.

The use of artificial neural networks allows for the determination of a mortar composition with a validation error of less than 5% and a method of classification of the type of mortar, which provides correct answers in more than 93% of cases. The characteristic values of the developed method for determining mortar type and composition prove its usefulness at construction sites, especially during conservation work. The methods presented in this paper are widely used in building materials laboratories and are described in European standards. Other methods, such as MIP or SEM-EDX, might be easily implemented in laboratories owing to the availability of such equipment, which gives a strong practical aspect to this work.

## Figures and Tables

**Table 1:** Elemental composition of compounds used for mortars.

Sample	LOI [%]	SiO_2_ [%]	CaO [%]	Al_2_O_3_ [%]	Fe_2_O_3_ [%]	MgO [%]	Na_2_O (%)	K_2_O (%)	SO_3_ (%)
cement	3.19 ± 0.06	18.84 ± 0.06	62.15 ± 1.16	5.08 ± 0.06	4.59 ± 0.05	1.65 ± 0.03	0.13 ± 0.01	0.55 ± 0.01	2.80 ± 0.07
lime	25.11 ± 0.06	1.52 ± 0.01	70.34 ± 1.32	0.50 ± 0.01	0.04 ± 0.01	0.50 ± 0.01	0.01 ± 0.01	0.05 ± 0.01	0.37 ± 0.01
sand	7.29 ± 0.06	75.05 ± 0.22	8.44 ± 0.16	4.02 ± 0.04	1.73 ± 0.02	0.86 ± 0.01	0.77 ± 0.01	1.34 ± 0.01	0.19 ± 0.01

**Table 2:** Composition of reference mortars.

**Cement Mortars**				
**Sample**	**Cement [g]**	**Sand [g]**		**Binder Content [%]**
A	500	1470		25.38
B	400	1770		18.43
C	300	1770		14.49
A1	500	1110		31.06
B1	400	1470		21.39
C1	300	1550		16.22
C2	300	1990		13.10
Cement–lime mortars				
Sample	Cement [g]	Lime [g]	Sand [g]	Binder content [%]
D	400	50	1770	20.27
E	300	70	1770	17.29
F	200	90	1770	14.08
G	150	140	1990	12.72
D1	400	50	2060	17.93
E1	300	70	2210	14.34
F1	200	90	2060	12.34
G1	150	140	2210	11.60
Lime mortars				
Sample	Lime [g]	Sand [g]		Binder content [%]
H	400	1930		17.17
I	300	1930		13.45
J	150	1930		7.21
H1	400	1280		23.81
I1	300	2890		9.40
J1	150	2410		5.86

**Table 3:** Results of mechanical properties tests.

Sample	Average Flexural Strength [MPa]	Average Compressive Strength [MPa]
A	5.18	29.35
B	4.03	17.76
C	2.94	10.77
A1	6.69	43.12
B1	4.03	21.52
C1	3.14	11.91
C2	2.37	8.09
D	4.20	19.21
E	3.40	12.10
F	2.15	6.25
G	1.34	2.51
D1	3.09	13.67
E1	0.52	8.78
F1	0.34	5.06
G1	0.16	2.64
H	-	0.13
I	-	0.11
J	-	0.09
H1	-	0.11
I1	-	0.09
J1	-	0.18

**Table 4:** Physical and chemical characteristics of reference mortars.

Sample	Insoluble Residues [%]	Total Porosity [%]	Specific Density [g/cm^3^]	SiO_2_ Content (%)	CaO Content (%)	Si/Ca Ratio	Type
A	68.47	16.5	2.6023	69.73	14.92	0.43	CM
B	75.13	22.4	2.6361	77.03	10.26	0.45	CM
C	76.73	26.3	2.6863	80.29	8.56	0.47	CM
A1	62.80	12.5	2.5990	66.66	17.38	0.33	CM
B1	69.92	18.5	2.6042	73.23	13.11	0.49	CM
C1	77.16	23.2	2.6479	79.60	9.22	0.47	CM
C2	80.56	25.3	2.6449	82.31	7.76	0.50	CM
D	72.33	22.2	2.6455	75.56	11.86	0.36	CLM
E	75.98	24.7	2.6533	77.75	10.27	0.31	CLM
F	79.68	25.8	2.6795	79.80	9.31	0.25	CLM
G	81.91	28.4	2.6964	82.24	8.20	0.21	CLM
D1	76.33	24.2	2.6213	78.01	10.56	0.42	CLM
E1	77.97	28.8	2.6132	80.05	8.85	0.44	CLM
F1	79.87	23.2	2.6806	80.84	8.56	0.37	CLM
G1	82.54	24.2	2.6533	82.78	7.72	0.32	CLM
H	78.41	29.9	2.6576	77.71	11.75	0.11	LM
I	84.05	35.8	2.6401	81.58	9.10	0.09	LM
J	88.13	37.5	2.7193	86.80	5.06	0.06	LM
H1	71.59	22.9	2.6136	72.63	15.54	0.07	LM
I1	85.75	22.2	2.7087	84.55	7.19	0.11	LM
J1	91.20	26.4	2.7232	88.08	4.13	0.23	LM

**Table 9:** Parameters of pre-selected CM-ANNs.

ANN Symbol	Learning Quality	Learning Error	Testing Error	Validation Error	Learning Algorithm	Error Function	Activation—Hidden Neurons	Activation—Output Neurons
1-MLP 6-6-1	0.9987	0.0364	2.6806	0.0343	BFGS 11	SOS	Exponential	Linear
2-MLP 6-4-1	0.9930	0.4664	0.0831	0.1843	BFGS 6	SOS	Linear	Tanh
3-MLP 6-4-1	1.0000	0.0007	0.7399	0.2095	BFGS 12	SOS	Exponential	Linear
4-MLP 6-10-1	0.9938	0.2141	0.1691	0.3967	BFGS 3	SOS	Tanh	Linear
5-MLP 6-4-1	1.0000	0.0000	4.9134	1.4852	BFGS 714	SOS	Tanh	Exponential

**Table 10:** Results of the sensitivity analysis of CM-ANNs.

ANN Symbol	SiO_2_ Content	CaO Content	Si/Ca Ratio	Specific Density	Total Porosity	Insoluble Residues
2-MLP 6-4-1	1.5144	6.7334	4.3065	0.9950	3.3892	2.2515
3-MLP 6-4-1	18.7058	5.5472	8.0104	9.8773	1,2859	1.0668
4-MLP 6-10-1	1.6978	3.0551	1.6165	1.7596	1.1381	1.8652

**Table 11:** Parameters of pre-selected LM-ANNs.

ANN Symbol	Learning Quality	Learning Error	Testing Error	Validation Error	Learning Algorithm	Error Function	Activation—Hidden Neurons	Activation—Output Neurons
1-MLP 6-10-1	0.9866	0.2416	5.71	1.7251	BFGS 3	SOS	Exponential	Linear
2-MLP 6-4-1	1.0000	0.0000	22.06	79.988	BFGS 83	SOS	Linear	Tanh
3-MLP 6-5-1	1.0000	0.0018	22.06	0.0195	BFGS 10	SOS	Exponential	Tanh
4-MLP 6-9-1	1.0000	0.0000	22.06	0.9128	BFGS 62	SOS	Linear	Logistic
5-MLP 6-11-1	1.0000	0.00002	36,105.20	0.7285	BFGS 0	SOS	Linear	Exponential

**Table 12:** Results of the sensitivity analysis of LM-ANNs.

ANN Symbol	Total Porosity	CaO Content	SiO_2_ Content	Insoluble Residues	Specific Density	Si/Ca Ratio
1 MLP 6-10-1	1.4289	0.8660	1.1428	1.1799	1.0587	0.2503
3 MLP 6-5-1	1.0064	1.0902	1.0616	1.0508	1.0181	1.0171
4 MLP 6-9-1	1.3092	1.5555	1.2829	1.0804	1.0728	1.0887

**Table 13:** Parameters of pre-selected CLM-ANNs (cement content).

ANN Symbol	Learning Quality	Learning Error	Testing Error	Validation Error	Learning Algorithm	Error Function	Activation—Hidden Neurons	Activation—Output Neurons
1-MLP 6-10-1	0.6039	6.8724	0.2652	28.4870	BFGS 1	SOS	Logistic	Exponential
2-MLP 6-8-1	0.9845	2.4044	0.1970	16.8200	BFGS 2	SOS	Logistic	Exponential
3-MLP 6-12-1	0.9934	0.0936	0.1366	2.2780	BFGS 5	SOS	Exponential	Logistic
4-MLP 6-8-1	0.5265	6.8993	0.2810	28.5740	BFGS 1	SOS	Logistic	Logistic
5-MLP 6-10-1	0.8828	6.8270	0.2330	28.5840	BFGS 1	SOS	Linear	Logistic

**Table 14:** Results of sensitivity analysis CLM-ANNs (cement content).

ANN Symbol	CaO Content	SiO_2_ Content	Insoluble Residues	Specific Density	Si/Ca Ratio	Total Porosity
2-MLP 6-8-1	1.1007	1.1313	1.1488	1.0628	1.0232	0.9945
3-MLP 6-12-1	3.0036	2.2041	1.9733	1.6411	1.3755	1.3907

**Table 15:** Parameters of pre-selected CLM-ANNs (lime content).

ANN Symbol	Learning Quality	Learning Error	Testing Error	Validation Error	Learning Algorithm	Error Function	Activation—Hidden Neurons	Activation—Output Neurons
1-MLP 6-5-1	0.9911	0.0242	0.0021	0.0314	BFGS 15	SOS	Linear	Exponential
2-MLP 6-8-1	0.9624	0.1029	0.0108	0.0338	BFGS 4	SOS	Exponential	Exponential
3-MLP 6-10-1	0.9659	0.1071	0.0010	0.0513	BFGS 3	SOS	Logistic	Exponential
4-MLP 6-6-1	0.9733	0.1161	0.0031	0.0560	BFGS 2	SOS	Tanh	Exponential
5-MLP 6-8-1	0.9686	0.1703	0.0062	0.1896	BFGS 3	SOS	Tanh	Tanh

**Table 5:** Characteristic of input data.

Performance	Insoluble Residues	Total Porosity	Specific Density	SiO_2_ Content	CaO Content	Si/Ca Ratio
Minimum (learning)	62.80	12.50	2.60	66.66	7.72	0.06
Maximum (learning)	84.05	35.80	2.70	82.78	17.38	0.50
Average (learning)	76.37	24.35	2.64	77.64	10.75	0.33
Standard deviation (learning)	5.90	5.66	0.03	4.93	3.09	0.15
Minimum (test)	77.16	23.20	2.65	79.60	4.13	0.23
Maximum (test)	91.20	37.50	2.72	88.08	9.22	0.32
Average (test)	85.50	29.03	2.70	84.83	6.14	0.27
Standard deviation (test)	7.38	7.50	0.04	4.57	2.71	0.05
Minimum (validation)	72.33	22.20	2.62	75.56	7.19	0.11
Maximum (validation)	85.75	24.20	2 71	84.55	11.36	0.49
Average (validation)	78.14	22.87	2 66	79.37	9.87	0.24
Standard deviation (validation)	7.23	5.13	0.05	7.05	3.70	0.26
Minimum (total)	62.80	12.50	2.60	66.66	4.13	0.06
Maximum (total)	91.20	37.50	2.72	88.08	17.38	0.50
Average (total)	77.93	24.80	2.65	78.92	9.97	0.31
Standard deviation (total)	6.71	5.62	0.04	5.27	3.27	0.15

**Table 6:** Pre-selected classification ANNs.

ANN Symbol	Learning Quality	Testing Quality	Validation Quality	Learning Algorithm	Error Function	Activation—Hidden Neurons	Activation—Output Neurons
1-MLP 6-4-3	46.67	66.67	33.33	BFGS 4	SOS	Logistic	Linear
2-MLP 6-5-3	46.67	100.00	33.33	BFGS 5	SOS	Tanh	Linear
3-MLP 6-5-3	93.33	33.33	33.33	BFGS 0	SOS	Tanh	Linear

**Table 7:** Summary of the classification matrix for the ANNs.

ANN Symbol	Result	Type-CM	Type-CLM	Type-LM	Type-All
1-MLP 6-4-3	correct (%)	66.6667	33.3333	33.3333	46.6667
	incorrect (%)	33.3333	66.6667	66.6667	53.3333
2-MLP 6-5-3	correct (%)	83.3333	0.0000	66.6667	46.6667
	incorrect (%)	16.6667	100.0000	33.3333	53.3333
3-MLP 6-5-3	correct (%)	100.0000	83.3333	100.0000	93.3333
	incorrect (%)	0.0000	16.6667	0.0000	6.6667

**Table 8:** Results of the sensitivity analysis.

ANN Symbol	Total Porosity	Insoluble Residues	SiO_2_ Content	CaO Content	Specific Density	Si/Ca Ratio
1-MLP 6-4-3	1.0191	1.0143	0.9978	0.9865	0.9997	1.0187
2-MLP 6-5-3	1.0139	0.9896	1.0000	0.9771	0.9912	1.0438
3-MLP 6-5-3	8.5702	6.9851	5.7865	5.0194	1.2098	0.8368

## Data Availability

The original contributions presented in this study are included in the article. Further inquiries can be directed to the corresponding author.
